# Whose Voice is it Anyway? *Artificial Intelligence and the New Crisis of Authenticity in Medical Education*

**DOI:** 10.5334/pme.2265

**Published:** 2026-04-01

**Authors:** Jessica Maher, Anna Byszewski, Heather Lochnan

**Affiliations:** 1 Faculty of Medicine, University of Ottawa, Canada; 2 Department of Medicine, Division of Geriatrics, The Ottawa Hospital, Canada; 3 Department of Medicine, Division of Endocrinology and Metabolism, The Ottawa Hospital, Canada

## Abstract

Generative artificial intelligence (AI) has entered the spaces of reflection and narrative learning in medical education, spaces once defined by authenticity, introspection, and human voice. This Eye Opener examines tensions identified through a recent survey of undergraduate medical students and their ePortfolio physician coaches at the University of Ottawa regarding the use of AI in reflective writing. While the student response rate was notably lower (9.6%) than that of coaches (52.9%), this asymmetry prompted consideration of how the evaluative context of reflective writing may influence disclosure of AI use in voluntary surveys.

Students who did respond described AI as a scaffold for idea generation and writing support, whereas coaches expressed unease, perceiving even limited AI use as a potential threat to authenticity. The resulting tension between pragmatism and preservation mirrors broader questions in medical education regarding reflective authorship and independent critical thinking.

This moment invites reconsideration of reflection not solely as a written product but as a developmental process. Educators may need to move beyond detection toward dialogue that supports psychological safety, transparency, and shared understanding of authorship in the age of digitally assisted writing. Reflection has long been understood as a means of engaging with professional identity formation. The challenge now is determining how this process can remain meaningful as generative tools become increasingly embedded in learners’ academic work.

## Setting the Scene: The Quiet Revolution

A medical student opens the ePortfolio platform, cursor blinking over a blank box requiring them to reflect on their professional growth. The prompt is familiar, the fatigue, too. This time, however, another voice lingers at the margins of the screen, the easy promise of ChatGPT.

Across the world, generative AI has become the invisible co-author of countless student essays, emails, and reflections [1,2,3,4]. Recent surveys demonstrate that 62 to 86% of medical students have used ChatGPT or similar AI tools for academic purposes, with primary applications including information searches, completing academic assignments, and academic writing [1,3,4,5,6]. ChatGPT has emerged as the most frequently used tool, typically discovered independently through social media or peer communication rather than institutional promotion [6,7]. In medical education, where reflection has long been considered sacred ground for meaning making and identity formation, its arrival is unsettling. Reflection has traditionally been a human act, an unfiltered conversation between experience and self. Yet, when machines begin to mediate that conversation, whose voice are we hearing?

The University of Ottawa’s ePortfolio program, developed and implemented in 2010, was designed to cultivate reflective practice through longitudinal mentorship between students and physician coaches [8]. Over four years, students post narrative reflections linked to Canadian Medical Education Directives for Specialists (CanMEDS) roles, and coaches respond, guiding both thought and growth [9]. The goal is not eloquence but authenticity. Yet, as AI tools became ubiquitous, new questions arose. How many reflections were still authentic? How would coaches know? What does authenticity mean when every sentence can be generated in seconds?

## When We Asked the Question

To explore these tensions, we conducted separate electronic surveys of undergraduate medical students and ePortfolio physician coaches at the University of Ottawa in 2024 to assess experiences, comfort, and attitudes toward the use of artificial intelligence in reflective writing.

Separate but parallel questionnaires were developed for student and coach participants to capture demographic information, experiences with AI use in the ePortfolio context, perceived norms surrounding AI utilization, and perspectives on institutional policy. Both surveys included a combination of multiple-choice items, five-point Likert scale questions, and optional free-text responses. Core items were designed to assess similar constructs across both groups while reflecting differences in participant roles within the ePortfolio program. All survey items administered to each participant group are provided in Appendix A, along with a mapping of corresponding constructs between student and coach questionnaires.

Eligible participants included all undergraduate medical students enrolled in Years 1 to 3 of the four-year MD program during the 2024 academic year, as well as all active ePortfolio physician coaches affiliated with the undergraduate medical education program at the time of survey distribution. Students in their final year of training were not invited to participate, as they were in the process of transitioning to residency during the survey period.

Survey invitations were distributed via institutional email to all eligible participants, with two follow-up reminder emails sent during the data collection period. Concurrent distribution of the survey link through student class group chats was undertaken to increase visibility. Participation was voluntary and responses were collected anonymously. Given the evaluative nature of reflective writing within the ePortfolio curriculum, anonymity was maintained to minimize social desirability bias and potential concerns related to disclosure of AI use. No identifying information was collected. This study was reviewed by the University of Ottawa Research Ethics Board and deemed exempt from formal Research Ethics Board review as a program evaluation initiative.

In total, 479 medical students and 70 physician coaches were invited to participate. Forty-six students (9.6%) and thirty-seven coaches (52.9%) completed the survey. Among student respondents (n = 46), representation spanned multiple cohorts within the undergraduate medical program (MD2025: 17.4%, n = 8; MD2026: 43.5%, n = 20; MD2027: 39.1%, n = 18). Among coach respondents (n = 37), duration of experience within the ePortfolio mentorship role ranged from one to over nine years (1 to 2 years: 27.0%, n = 10; 3 to 4 years: 18.9%, n = 7; 5 to 6 years: 10.8%, n = 4; 7 to 8 years: 13.5%, n = 5; 9 or more years: 29.7%, n = 11).

At our institution, reflective writing within the ePortfolio curriculum forms part of a formally reviewed professional development process. It is possible that concerns regarding potential disclosure of AI use within an evaluative educational context influenced willingness to participate, even in the absence of identifiable data collection. Institutional data on voluntary survey response rates among undergraduate medical students are not routinely tracked, and as such, we were unable to evaluate this response rate in comparison with surveys addressing less sensitive topics or those for which students may perceive fewer potential academic or professional repercussions. As such, non-response was not treated as a coded qualitative finding but rather as a contextual feature of participation that informed the interpretation of survey responses.

Responses were analyzed on an item-by-item basis. Missing responses were excluded from analyses for the specific item to which they pertained, without imputation. As such, item-level response variability was anticipated and interpreted as reflecting differences in participant comfort with disclosure across question domains.

Free text survey responses, available in Appendix B, were analyzed using reflexive thematic analysis as described by Braun and Clarke [10]. This approach was selected to support interpretive exploration of participant meaning-making rather than measurement of response prevalence.

## The Meaning of the Silence

In a curriculum where reflective writing is reviewed by faculty as part of professional development assessment, authenticity has always carried some degree of evaluative risk. The emergence of generative AI may have made these tensions more visible. Students may be uncertain how the use of such tools will be interpreted by coaches or faculty, or whether disclosure could prompt increased scrutiny of reflective submissions. Within this context, reluctance to participate in a survey addressing AI use may reflect concern about being identified as relying on external assistance, even when responses are anonymized.

Student Participant 7 captured this tension succinctly:

*“I think students need to come up with the content to be a genuine reflection but can use AI as an assistant to put it into words.”*

Coaches, however, saw such assistance as dilution. Coach Participant 21 noted:

*“The whole purpose of ePortfolio is for students to personally reflect on components of their training… and to do it themselves.”*

## Two Ways of Seeing

Students tended to view AI as a pragmatic tool. Student Participant 21 wrote:

*“AI could be used to brainstorm ideas when students are having difficulty coming up with a topic or idea to write about.”*

In another vein, Student Participant 32 reflected:

*“It helps to make ideas come to life in a beautiful way… I think AI is great to enrich yourself and to learn and make your thoughts clear!”*

For Coach Participant 34, however, the same technology represented erosion of meaning:

*“If the students are using AI to generate the post… why bother? It’s a meaningless exercise then and a waste of my time as a coach.”*

## Beyond Policing: Reclaiming Reflection as Dialogue

The instinct to regulate AI use, to craft policies, deploy detectors, or issue penalties, is understandable but misdirected. Students and coaches alike reported minimal awareness of any institutional AI policy. Coach Participant 8 advocated:

*“There should be specific rules of engagement… not appropriate for students to rely on AI to create their composition from scratch.”*

Yet even Coach Participant 10 acknowledged limits:

*“I don’t think I should have any role in this… truth be told, we are not trained on how to screen for AI usage.”*

In contrast, several students emphasized autonomy. Student Participant 19 argued:

*“If a student feels like AI can accelerate their thinking or writing process, they should be free to use it as a tool.”*

## Re-imagining the Narrative Curriculum

The rise of AI invites reconsideration of what reflection is intended to accomplish within undergraduate medical education. If the goal is polished prose, generative tools will likely outperform learners. If the goal is insight, empathy, and professional identity formation, the reflective process must remain grounded in personal meaning making, even when digitally assisted.

As Coach Participant 10 conceded:

*“There is no question AI will be used increasingly by students… rather than fighting it, embracing it is probably the way to go.”*

This shift may also require reconsideration of how reflection is scaffolded and assessed within undergraduate medical curricula. If the presence of AI is inevitable, educational approaches that emphasize process over product may better preserve the developmental intent of reflective practice. For example, greater emphasis on longitudinal dialogue with coaches, verbal or in-person reflective conversations, or iterative submissions that capture the evolution of student thinking over time may offer alternative means of assessing growth that are less dependent on written prose alone. Rather than positioning AI as inherently incompatible with reflection, educators may need to clarify expectations regarding appropriate use, transparency, and authorship, while ensuring that assessment strategies continue to privilege personal meaning making over linguistic polish.

## Conclusion: Holding the Mirror

Our findings underscore a new tension in medical education. Not because AI writes reflections, but because it may highlight divergent or shifting understandings of authenticity among learners and educators within reflective curricula.

Perhaps the more pressing question is not whether students are using AI, but how educators wish reflection to function in an era where writing can be digitally assisted. If reflection is meant to cultivate insight, vulnerability, and professional identity, then assessment structures must support psychological safety rather than surveillance. This may include explicitly defining acceptable uses of generative tools within reflective work and prioritizing coaching conversations that examine the thinking underlying a submission rather than the linguistic features of the text itself.

As practices of documentation continue to evolve, reflection remains a central mechanism through which learners engage in professional identity formation. In the age of generative AI, the more relevant question may be less whether a reflection was written independently and more whether it authentically represents a learner’s thinking, effort, and development.

## Additional Files

The additional files for this article can be found as follows:

10.5334/pme.2265.s1 Appendix A. Student and Coach Survey Instruments with Cross-Population Item Mapping.

10.5334/pme.2265.s2 Appendix B. Complete Verbatim Free-Text Survey Responses from Student and Coach Participants Supporting the Qualitative Analysis.

## AI Disclosure

ChatGPT (OpenAI) was used to assist in the organization of qualitative free text responses and for proofreading and stylistic recommendations during preparation of the final draft. All content, interpretation, and revisions were performed and verified by the authors, who take full responsibility for the manuscript’s accuracy and integrity.
