# Supplementary material for: Whose Voice is it Anyway? Artificial Intelligence and the New Crisis of Authenticity in Medical Education
Source: Perspect Med Educ. 2026 Apr 1;15(1):351–5. doi: 10.5334/pme.2265 (PMC13045798; doi:10.5334/pme.2265)
Supplement: Appendix A. — Student and Coach Survey Instruments with Cross-Population Item Mapping. [file pme-15-1-2265-s1.pdf]

Appendix A. Student and Coach Survey Instruments with Cross-Population Item Mapping

Separate but parallel questionnaires were administered to undergraduate medical students and ePortfolio physician-coaches. Core items were designed to assess similar constructs across both groups, while additional role-specific questions were included where appropriate. Corresponding items between surveys are mapped below. Items unique to either population are indicated as such.

Student Survey Instrument

- Q1. What is your anticipated year of graduation? (Student-specific demographic item)
- Q2. Which of the following describes your usage of AI in ePortfolio exercises? (Select all that apply)
- Q3. If you have used AI for ePortfolio exercises, please list all types of AI used (eg. ChatGPT, Copilot, etc.). (Student-specific)
- Q4. If you have used AI for ePortfolio exercises, please describe your reason for doing so (150 word limit). (Student-specific)
- Q5. Agreement statements regarding comfort with AI, familiarity with expectations, citation of AI use, and alignment with other UGME tasks.
- Q6. Agreement statements regarding professionalism, AI as a tool, referencing, plagiarism, monitoring, consequences, and advantage.
- Q7. Awareness of University of Ottawa AI policies.
- Q8. Awareness of Faculty of Medicine/UGME AI policies.
- Q9. Belief in need for policies governing AI usage.
- Q10. Purpose of ePortfolio and role of AI (150 word limit).
- Q11. Governance recommendations for AI usage (150 word limit).
- Q12. Use of AI in other academic contexts. (Student-specific)
- Q13. Comparison of AI use inside vs outside ePortfolio. (Student-specific)
- Q14. Description of AI use in other contexts (150 word limit). (Student-specific)
- Q15. Willingness to participate in focus group.

Coach Survey Instrument

- Q1. How long have you been an ePortfolio coach? (Coach-specific demographic item)
- Q2. Understanding of students' usage of AI in ePortfolio exercises.
- Q3. Agreement statements regarding comfort, familiarity, citation expectations, alignment with UGME use, and monitoring. (Monitoring item is coach-specific)
- Q4. Agreement statements regarding professionalism, AI as a tool, referencing, plagiarism, monitoring, consequences, and advantage.
- Q5. Awareness of University of Ottawa AI policies.

- Q6. Awareness of Faculty of Medicine/UGME AI policies.
- Q7. Belief in need for policies governing AI usage.
- Q8. Governance recommendations for AI usage (150 word limit).
- Q9. Purpose of ePortfolio and role of AI (150 word limit).
- Q10. Role in monitoring or initiating disciplinary actions (150 word limit). (Coach-specific)
- Q11. Observed changes in AI usage over time. (Coach-specific)
- Q12. Description of observed changes (150 word limit). (Coach-specific)
- Q13. Impact on motivation to continue coaching (150 word limit). (Coach-specific)
- Q14. Willingness to participate in focus group.

Cross-Population Item Mapping

Construct	Student Survey Item(s)	Coach Survey Item(s)
AI usage in ePortfolio	Q2	Q2
Comfort with AI usage	Q5	Q3
Familiarity with expectations	Q5	Q3
Referencing AI contributions	Q5	Q3
Alignment with other UGME AI use	Q5	Q3
Professional standards	Q6	Q4
AI as academic tool	Q6	Q4
Referencing requirements	Q6	Q4
AI as plagiarism	Q6	Q4
Monitoring for AI use	Q6	Q3, Q4
Consequences for AI use	Q6	Q4
AI policy awareness	Q7, Q8	Q5, Q6
Need for AI policy	Q9	Q7
Role of AI in ePortfolio	Q10	Q9

Governance recommendations	Q11	Q8
Focus group interest	Q15	Q14
