# Supplementary material for: Whose Voice is it Anyway? Artificial Intelligence and the New Crisis of Authenticity in Medical Education
Source: Perspect Med Educ. 2026 Apr 1;15(1):351–5. doi: 10.5334/pme.2265 (PMC13045798; doi:10.5334/pme.2265)
Supplement: Appendix B. — Complete Verbatim Free-Text Survey Responses from Student and Coach Participants Supporting the Qualitative Analysis. [file pme-15-1-2265-s2.pdf]

Appendix B. Complete Verbatim Free-Text Survey Responses from Student and Coach Participants Supporting the Qualitative Analysis

Coach Respondent Number	Q8 Response – "Please describe what you believe should be in place, if anything, to govern AI utilization by students in the ePortfolio context?"	Q9 Response – "What do you believe is the purpose of ePortfolio, and what role could/should AI play in fulfilling this purpose?"	Q10 Response – "What do you believe your role should be in monitoring for AI utilization by students and/or initiating disciplinary actions, where applicable?"	Q13 Response – "How has the utilization of AI by students in ePortfolio exercises impacted your motivation to continue coaching, if at all?"
**1**	"I think AI can be used but quotations are mandatory. We should be telling our students that those are the rules."	"eportfolio is a means of journaling emotions and exploring experiences through medicine, which is important for life long resiliency"	"It will be hard for any coach to monitor for this on top of the million other things we do - ultimately, it will be the student who loses out if they are not journaling their true emotions or experiences."	It hasn't impacted my motivation to continue as a coach at this point but I have only done it for 1 year.
**2**	"I think it's all blown out of proportion, and students can use AI as tool"	"AI can speed things up, but a student still needs to be sure that what is written is what they are thinking, a reflection is hard to write with AI as it may be not your thinking anymore."	"it's a good topic to discuss with the group and see what the consensus is for the students"	"no change"
**3**	Respondent skipped this question	"The purpose of ePortfolio is for help students develop as professional and as people, AI should not be part of this"	Respondent skipped this question	"Yes, I am less interested in ePortfolio exercises when the students use AI"
**4**	"I do not see a role for AI in the eportfolio."	"Purpose if for self asssesment and reflexion on the different role to become a competent professional clinician, I do not see a role for AI"	"I would need to know what are the U of O policies on AI use in eportfolio; i am rather clueless about AI and how i would go about monitoring its utilization."	"Should AI be allowed to be used in eportfolio, it should be clearly identified by the student and there should be a clearly identified personal reflexion/self assessment to complement."
**5**	"I'd like to know the recommendations for AI use from UGME"	"Don't know about AI use in ePorfolio"	"Don't know and I don't know how to monitor for AI use"	"Haven't thought about it yet"
**6**	"AI has no place in academic setting"	"EPortfolio is a time of reflection. It is not a chore. The words chosen are very important. I am very familiar with AI as I use it for patient encounters and I know that the text created by AI has content but is scripted and does not translate well more complex thoughts."	"Should be the faculty's role to have policy and policing processes in place."	"I am not aware of AI use in my group. I will be enquiring."
**7**	Respondent skipped this question	Respondent skipped this question	Respondent skipped this question	Respondent skipped this question

**8**	"There should be specific rules of engagement on the extent of AI utilization. For example, I don't think it's appropriate for students to rely on AI to create their composition from scratch. I think that while ePortfolio submissions are of lower consequence because coaches are not necessarily evaluating students based on writing ability, the implications of allowing AI to complete 90% of a project for university thesis projects has strong implications for student evaluation"	"ePortfolio is meant to create a space for individual reflection and dialogue. The goals can be broad ranging, but can be a platform for anything from self-evaluation towards a learning objective, or on a bigger scale, a platform for journaling career directions, for example."	"To some extent, coaches should pick up on very obvious cases of heavy AI usage, but truth be told, we are not trained on how to screen for AI usage, nor are we trained on how to resolve these dilemmas. Nor have we been told about the chain of reporting and documentation of such events."	"I have not detected AI usage so far."
**9**	"I doubt we can put anything in place that would be enforceable. All we can do is emphasize that the purpose of ePortfolio is to foster reflection. The exercise has no value if students don't write the posts in the conventional way."	"I think AI is antithetical to the purpose of ePortfolio, which is to foster reflection. But I think that cheating on your ePortfolio is like cheating on your diet: in the end you're only cheating yourself."	"None. Students are grown ups, and ePortfolio is not such an intergral portion of the curriculum that there should be consequences for doing a crummy job with it. To repeat my comment above, cheating on eportfolio is like cheating on your diet: you're only really cheating yourself."	"It honestly never occured to me until I got this survey that students would be using it, though it now seems obvious. At first blush, it is sapping my motivation."
**10**	"There is no question AI will be used increasingly by students in ways that I (even as a relatively tech-savvy 35-year-old) cannot fathom. Rather than fighting it, embracing it is probably the way to go. Even in creating the prompts for an AI generated piece, and going through the iterative process there is probably some reflective value."	"ePortfolio purpose: 1) Have structured reflection on things that students go through in their medical school time. 2) Practice reflecting for when it is less mandated later in career. 3) Provide repository of interactions/events/encounters for reference when preparing for residency applications. Role of AI: variable based on student's comfort using it."	"I don't think I should have any role in this. If students use AI and I can tell there has been no reflection or intentional thought on their part, that will show in their work and I will comment on it."	"None - this is the way the world is going."
**11**	Respondent skipped this question	Respondent skipped this question	Respondent skipped this question	Respondent skipped this question
**12**	Respondent skipped this question	"Aucun role" [Translated: "No role."]	"Dur à renforcer" [Translated: "Difficult to reinforce."]	"Si j'apprends que mes étudiants utilise l'AI, je serai découragée à l'enseigner" [Translated: "If I learn that my students use AI, I would be discouraged from teaching it."]
**13**	"I don't think it should be used. Use might generate a post meeting the stated requirements but the student will not have achieved the learning goals. Goal isn't only the product- the	"I think the goal is to describe and reflect on various medical student experiences and discuss them within the different roles and objectives of eportfolio and learn the	"None. It should not be used and there should be no role for the coach to monitor for use of AI. This should fall to the administration to monitor if required."	"I don't know if it is being used. If so it is not being cited."

	posts but the process of writing and reflecting that results in the post. The goal is to develop reflection skills. You don't use a calculator prior to learning basic math skills- if you want to use the analogy from an earlier question"	foundational skills of self reflection that will be used for your medical career"		
**14**	Respondent skipped this question	Respondent skipped this question	Respondent skipped this question	Respondent skipped this question
**15**	Respondent skipped this question	"The purpose is choosing a learning point from a case and reflecting on it and sharing it with others. Ai could help to write it up."	Respondent skipped this question	Respondent skipped this question
**16**	"Rules/guide on use. Certainly it should be referenced. Would need to discuss whether we could consider that it may play some helpful role. I would feel though that completely AI generated posts make the written component of the task a bit pointless. So then should it be group sessions without questions to prepare in advance."	"Students would still need to consider an encounter/event and present AI software with some themes to explore (to my knowledge of the technology) & we could consider that what is generated may prompt further reflection for the student. However this should definitely be disclosed and again I feel it places more emphasis on the group sessions"	"I would not wish to take on checking/disciplinary role. As adult learners I expect medical students to have the responsibility/respect to complete a tasks as requested. AI clearly new player in academia so need rules around its use."	"Think greater importance then of meetings for discussion & reflection"
**17**	Respondent skipped this question	Respondent skipped this question	Respondent skipped this question	Respondent skipped this question
**18**	"As the entire purpose of eportfolio is personal reflection the role in this setting should be limited (ideally to grammar and spelling etc if that is "AI" use to build content defeats the purpose."	"as above, the purpose of this program is to have learners reflect on their own personal experiences. It is hard to see a role for AI"	"It is a sad thought that we would need to check for AI use, but we likely need some training to learn how to do this."	"Until this survey, I had not actually considered that the students would use AI to generate eportfolio posts given the personal nature. If this was broad practice beyond very basic elements, would not see the point of having the program at all."
**19**	Respondent skipped this question	Respondent skipped this question	Respondent skipped this question	Respondent skipped this question
**20**	Respondent skipped this question	Respondent skipped this question	Respondent skipped this question	Respondent skipped this question
**21**	"The whole purpose of ePortfolio is for students to personally reflect on components of their training - and to do it themselves."	"AI should not have a role, it should be explicitly banned."	"None - I will resign from being an ePortfolio coach if that is an expectation. Being an ePortfolio coach is a joy. Being a police officer /academic honesty investigator is not!"	"As above - I have no idea, students are not telling me they are using AI"
**22**	Respondent skipped this question	Respondent skipped this question	Respondent skipped this question	Respondent skipped this question
**23**	"AI use should be banned in e-portfolio and the consequences of use of AI should be the same as cheating on an exam or plagiarism."	"AI use in e-portfolio is counter to the goal"	"The university needs to develop a process/tool to discern which posts are likely AI generated."	"If students disengage from the point of e-portfolio (which is self-reflection, not AI-generated reflection) there is no point having a mentor."

**24**	"I struggled with your second question as I had not considered the use for self reflection."	"self reflection"	"As this is self reflection and there has been evidence showing that not performing self reflection leads to more complaints in future clinical practice I think it is up to the student."	"has not changed for me yet"
**25**	"AI use must be banned in the ePortfolio and academic context in general"	"The purpose of ePortfolio is self-reflection. IA contravene with this."	"Coaches should flag AI usage to the faculty."	"AI use defeats the purpose of ePortfolio."
**26**	"I don't know enough about AI to comment. I think we should ask the experts (likely the students)."	"Recording experiences to reflect on throughout training and creating a bank that can be drawn from during CaRMS interviews."	"I don't know enough about AI usage and how it might be harmful. I think if the med student is generating the ideas from which the writing comes and curating the post, they can benefit from it."	"N/A"
**27**	"Prefer no AI. If AI used, it should be stated that it was used and why it was used."	"Self reflection on the roles of an MD is the purpose of e-portfolio. AI could provide a starting point for deeper reflection."	"None."	"No, I had never considered that it was used or that it was a problem (maybe naively)."
**28**	"Guidelines regarding when its use is allowed and when it is not. Would be okay to use AI to generate ideas however there should still be personally written content."	"Role is for students to have a structured opportunity to reflect on their experiences. Ultimately students will put as much effort as they personally see fit and mandating different rules is not likely to change their commitment to it. For this reason I certainly don't think AI should be banned. Students who find a way to use it to their benefit should be allowed."	"I don't think there should be a role for coaches to surveil AI usage. Should be based on honour system."	"Has not changed"
**29**	"Not sure AI should be used for portefolio although I think it has a place in other parts of the medical curiculum. The reason for portefolio is to allow reflection on the human connection in medicine - not a place for AI"	"Don't think AI has a role in portefolio unless one is using it to organize ones thoughts"	"If AI. Id used to get away with the initial purpose of portefolio , which is to reflect on our actions as physicians , then I think that needs to be addressed. However , don't think it is my role to start policing students about inappropriate use of AI once policies have been put in place on how it should be used"	"Not aware students were using so can't comment"
**30**	"There should be an automatic detection of AI usage in ePortfolio."	"The students need to discuss their own real life experiences and how they can grow from these things. We talk about introspection. If AI is doing the work, there is a	"I am not sure about this. ePortfolio has encouraged artistic and alternative posts, so AI could potentially be used if the student has done the reflection."	"No."

		concern about a lack of genuine experience and reflection on the part of the student.”		
**31**	Respondent skipped this question	Respondent skipped this question	Respondent skipped this question	Respondent skipped this question
**32**	“Do not allow the usage”	“Reflective of one’s experience. Writing it out helps student going through the thoughts and emotions.”	“Tell students not to use it.”	Respondent skipped this question
**33**	“The point of ePortfolio is to foster self reflection and critical thinking. AI should not be used to facilitate this.”	“AI should have no role in ePortfolio. The platform submission process should integrate plagarism and AI checks similar to journal submissions. If this can't be undertaken the format of eportfolio may need to be modified.”	“None, the submission platform should be able to assess this. If coaches are required to check, training will need to be provided.”	“I would stop coaching. I want to help trainees learn how to reflect and prepare them from the challenges of practice that difficult situations can create.”
**34**	“I think some students are using AI for posts. I think if used, it should at the minimum be referenced.”	“E portfolio is to reflect on the experiences in medicine and learn about topics such as advocacy, professionalism, communication. I’m not opposed to AI proofreading post but if the students are using AI to generate the post - why bother? It’s a meaningless exercise<br>then and a waste of my time as a coach. I actually found myself considering quitting as a coach as reviewing the obvious AI<br>generated posts was such a waste of my time. I’ve enjoyed exploring the real experiences the students are having but if it’s going to turn into reading flowery AI written platitudes, I don’t see the point.”	“Given what you pay is to do this combined with the expectation that the students have that I’ll treat them to supper as well - nothing.<br>I’d rather just step away from coaching in this case.”	“I’m not interested in continued coaching in this case.”
**35**	“Il devrait faire mention que l'utilisation de l'AI pour des travaux de réflexion n'est pas encouragée car elle n'amène pas à une réflexion personnelle, ce qui va à l'encontre de l'objectif visé par ePortfolio.” [Translated: “It should be stated that the use of AI for reflective assignments is not encouraged, as it does not lead to personal reflection, which goes against the objective of the ePortfolio.”]	“Faire preuve de réflexion PERSONNELLE sur le développement de leurs compétences en tant que futurs professionnels de la santé.<br>L'utilisation de l'IA paraît contradictoire avec cet objectif.” [Translated: “Students should demonstrate PERSONAL reflection on the development of their competencies as future health professionals. The use of AI seems contradictory to this	“Je ne pense pas que ce soit le rôle du coach dans cette surveillance. Cette responsabilité devrait incomber aux étudiants, question de professionnalisme.” [Translated: “I do not think it is the coach’s role to monitor this. That responsibility should rest with the students themselves, as a matter of professionalism.”]	“Diminue la valeur de notre rôle.” [Translated: “It diminishes the value of our role.”]

		objective."]		
**36**	"Students should reference it's use and mentors/teachers should be given direction on how to scan or screen bodies of work for it."	"EPortfolio helps students reflect on their clinical experiences so that they can think critically about patient care, communication, professionalism and how their own feelings and beliefs can affect their work as a doctor."	"I think it falls in the category ensuring students are professional and ethical in their work."	"I am not currently coaching"
**37**	Respondent skipped this question	Respondent skipped this question	Respondent skipped this question	Respondent skipped this question

Student Respondent Number	Q4 – "If you have used AI for ePortfolio exercises, please describe your reason for doing so"	Q10 – "What do you believe is the purpose of ePortfolio, and what role could/should AI play in fulfilling this purpose"	Q11 – "Please describe what you believe should be in place, if anything, to govern AI utilization by students in the ePortfolio context?"	Q14 – "How do you use AI in other academic contexts? Please explain."
**1**	"Proofreading and editing"	Respondent skipped this question	Respondent skipped this question	"Generating ideas to guide my brainstorming process"
**2**	Respondent skipped this question	Respondent skipped this question	Respondent skipped this question	Respondent skipped this question
**3**	Respondent skipped this question	"The purpose of the portfolio is to encourage students to reflect upon the CANMeds roles and their connections to their expériences. In my opinion, AI does not have a place in this process as it substituâtes all the time students would otherwise invest in working on artiuclating their own opinions and expériences. It removes any créativité from the composition and turns a réflective exercice into one of inputs and outputs."	"I do not think the use of AI should be permitted in this context."	Respondent skipped this question
**4**	"It helps me come with ideas and ways of formatting my posts"	Respondent skipped this question	Respondent skipped this question	Respondent skipped this question
**5**	"it can help with creating"	"student reflection on the canmed roles, AI can be used to help students tailor their experience to one particular role, but shouldn't be used to generate the whole post."	"do a quick screen of the text to see if there's any AI generated text, while being mindful that many of these tools may be inaccurate, and that additional investigation may be warranted if >80% or some other high threshold is reached"	"idea generation given a prompt, study note explanations"
**6**	"N/A"	"Purpose of Eportfolio is to reflect upon our practice to improve on it and learn from our mistakes and others' and to encourage ethical	"As Eportfolio is a reflection and is very subjective to each individual I don't believe there should be any AI governance as it can be used to	"Utilities it to brainstorm ideas and to organize my schedules and improve on my knowledge."

		practice to uphold our responsibilities towards the public and our colleagues.”	proofread and that is no different from having someone else proof read the reflections.”	
**7**	Respondent skipped this question	ePortfolio is to reflect on clinical experiences. AI could help with the writing part. I think students need to come up with the content to be a genuine reflection but can use AI as an assitant to put it into words/writing because that can be hard and is not the purpose of ePortfolio (i.e. to learn to write). Since it's not published or a work that we take credit for in the public space (like it's not a research work for example), I don't think we need to cite AI or that it's "plagiarism" in this specific context.	Respondent skipped this question	“Learning/studying by asking questions to AI, writing emails, brainstorming ideas. I don't really use AI very often.”
**8**	“Just for editing purposeds”	“It's a way of self reflecting and AI can help make that efficient but it should be your own ideas”	Respondent skipped this question	Respondent skipped this question
**9**	Respondent skipped this question	“To me, I see ePortfolio as a platform that is intended to document the otherwise intangible character/professional growth of medical students. What might have otherwise been a conversation over coffee with a fellow student or a trusted preceptor is transformed into a digital item that is designed to (quite literally) check the boxes pertaining to CanMEDS roles, while still serving as material for those conversations via ePortfolio mentors. As to the role of AI, I think the underlying question is thus - "what benefit does ePortfolio in its current form provide medical students, and at what point along the automation gradient (i.e. using AI to write an outline vs. the entire passage) is this benefit meaningfully reduced?"”	“Truthfully, I do not know. Putting aside the institutional factors that might play into such a decision, I feel that it's ultimately going to first boil down to a question of enforcement; is it actually possible to enforce a policy on AI utilization with good sensitivity/specificity? As far as I am aware, there are currently no tools that can reliably both detect AI written articles and reliably exclude authentic ones.”	“I occasionally use ChatGPT in order to explain concepts that I find difficult to understand to me, and very rarely to help me draft an outline for writing emails.”
**10**	Respondent skipped this question	Respondent skipped this question	Respondent skipped this question	Respondent skipped this question
**11**	Respondent skipped this question	“Unclear. The exact purpose of	“AI use may help students organize	Respondent skipped this question

		ePortfolio is not well-understood. Its value may be more visible as time goes on. AI can help students generate writing ideas when they are under time stress.”	ideas and be more efficient. Since the ideas are still students' own, it is not an issue.”	
**12**	Respondent skipped this question	“The role of eportfolio is to help students reflect on their experiences and how they fit in canmed roles. While AI can't know your experiences, it could provide some inspiration for writing. It could help some, but I personally have not used it and can still see that the objectives could be met with its usage. However, the reflection should still require some thought on the writer's part.”	“Some guidance on how and when it can be used should be provided by the faculty”	Respondent skipped this question
**13**	“Able to transform my ideas in a nice story in a short amount of time”	“It helps me think of experiences related to the canned roles which may be helpful for Carms interviews.”	“I think that students should be allowed to use chatgpt freely. In every case, it is the students ideas that are being used. Chatgpt is just a tool like anything else that facilitates our work especially with our busy schedules.”	“I also use it for emails.”
**14**	“Only used it once due to lack of time, but it actually costed me more time and then it left me more mad afterwards… believe it or not that was the only time I’ve used it and all other times I’ve handwritten them!”	“Eportfolio will help me for carms and I think AI can help a bit with idea generation.”	“AI checkers aren’t too valid as of yet. But honestly it’s easy to tease out the difference between a real reflection vs an AI one. The one ChatGPT wrote for me sounded horrendous and very fake and just generic without you know… real life details. If someone used AI entirely to write it then I feel like it would be pretty apparent.”	“Everything”
**15**	Respondent skipped this question	“student reflection - therefore AI could make creating reflections easier, probably less of a problem for students to use AI in ePortfolio compared with other academic assignments (ie. SIM essays, etc.)”	Respondent skipped this question	“French translations for interest groups, creating medical cases to study from for PSD”
**16**	Respondent skipped this question	“AI can help generate ideas”	Respondent skipped this question	Respondent skipped this question
**17**	Respondent skipped this question	“Purpose is to reflect and using AI would not allow for good reflection”	Respondent skipped this question	Respondent skipped this question
**18**	Respondent skipped this question	Respondent skipped this question	Respondent skipped this question	Respondent skipped this question

**19**	"To speed up the brainstorming process"	"Purpose of ePortfolio: To drive reflection surrounding experiences in medicine. | Usage of AI: Whatever the student believes is helpful in their reflection/writing process."	"I don't know that anything is necessary. If a student feels like AI can accelerate their thinking or writing process, they should be free to use it as a tool."	"Usually to help brainstorm or sift through large amounts of data (i.e., generate summary). In my technology-related activities, I also use it to help guide or clean-up my coding."
**20**	Respondent skipped this question	"ePortfolio exercises are personal reflections to develop a repository of experiences related to the CANMeds Roles over the four years. While I have not used AI for these reflections, I have used the generated templates/suggestions for other personal written documents. As long as the writer can accurately convey their intent and modify any unclear/ambiguous phrases, AI will provide a great starting point for these posts. Care should be taken to not over-embellish or include personal/patient identification information in the software. It is the user's responsibility to ensure that the final output, or submitted document, meets the objectives and is truthful and accurate.<br>Just as physicians are using AI to complete patient charts, this is another resource to help reduce the administrative burden on medical students."	"In the spirit of full transparency, there is a growing dislike for the entire Eportfolio experience. It is one of many administrative tasks added to students and significantly complicates scheduling. Most colleagues have verbalized that they "make something up" and "we just gotta get through this meeting. " Given this context, I think that AI adds depth to responses that would not otherwise be included and prompt additional personal reflection. The most imperative question is not the regulation of AI in portfolio assignments but the perceived/actual benefit to the entire Eportfolio aspect of the curriculum."	"I use AI to assist with the generation of the necessary documents for the many extracirricular teams I am involved in or to assist with generating professional emails to the faculty."
**21**	"N/A"	"Reflecting on clinical experiences and growth in different domains as students progress through their medical training. AI could be used to brainstorm ideas when students are having difficulty coming up with a topic/idea to write about to meet a specific e-Portfolio CanMEDS role"	"I don't believe posts should be written up with AI assistance, however, as the whole purpose behind these posts centers around personal reflection - something that takes place actively during the drafting of the post."	"Mostly as a supplement to reinforce/facilitate understanding of more complex topics being taught in the curriculum (ex. obtaining an easy-to-understand summary of the main differences between obstructive vs. restrictive lung disease)"
**22**	Respondent skipped this question	"ePortfolio is meant to be an exercise in personal reflection, and AI usage essentially makes that function useless. I understand the temptation to use shortcuts because ePortfolio can feel like just one more box-	Respondent skipped this question	"I have used ChatGPT to organize point form notes into a summarized version"

		ticking exercise."		
**23**	"It helps overcome writers block."	"I don't know the purpose of ePortfolio. It seems just like an opportunity to reflect on something and quickly share it with people who are just as tired as you are."	"Nothing"	Respondent skipped this question
**24**	Respondent skipped this question	Respondent skipped this question	Respondent skipped this question	Respondent skipped this question
**25**	Respondent skipped this question	"nous permettre de réfléchir sur nos expériences, nos attitudes, nos sentiments. AI pourrait nous aider à formuler nos idées, à bonifier notre entrée, à générer des idées de sujets sur lesquels rédiger" [Translated: "Allowing us to reflect on our experiences, our attitudes, and our feelings. AI could help us articulate our ideas, enhance our entry, and generate ideas for topics to write about."]	"Apprendre aux étudiants comment bien l'utiliser pour en tirer le maximum de profit. Pas nécessaire de restreindre et de mettre des réglementations car AI est un OUTIL qu'il faut apprendre à bien utiliser. Les gens trouveront une façon de contourner les règles de toute façon." [Translated: "Teaching students how to use it properly in order to get the maximum benefit from it. It is not necessary to restrict or impose regulations, because AI is a TOOL that must be learned and used well. People will find ways to get around rules anyway."]	"Traduction FR vers ENG" [Translated: "Translation from French to English."]
**26**	Respondent skipped this question	"None - we are supposed to come up with our own experiences not use AI to generate one for us"	"No clue."	Respondent skipped this question
**27**	"Insufficient time & energy to write them"	"Self reflection"	"Nothing"	Respondent skipped this question
**28**	Respondent skipped this question	"It's meant to be an area of personal reflection. Since AI cannot fake an experience, I believe any use of AI would just help the articulation of that experience and lesson"	"nothing"	Respondent skipped this question
**29**	"Efficiency and creativity"	"Reflect and prepare for interviews"	"None"	"Studying clinically everything"
**30**	Respondent skipped this question	Respondent skipped this question	Respondent skipped this question	Respondent skipped this question
**31**	"Mind ur own business"	"Mind ur own business"	Respondent skipped this question	Respondent skipped this question
**32**	"It helps to make idées come to life in a beautiful way. I think AI is great to enrich yourself and to learn and make your thoughts clear!"	Respondent skipped this question	"None"	Respondent skipped this question
**33**	"I find that the clarity of my statements often gets lost because I use too many filler words (due to a lack of writing in recent years). I	"I think that ePortfolio is a reflecting tool for students. It assists with providing us examples of CanMed roles for when we reach 4th year and	"I think its ok to use AI for brainstorming purposes, maybe to give you're an organization of your text and to proofread it (or even	"I use it to help clarify some of my written work (SIM presentations, etc) and to brainstorm at times. I do not use it as an information tool like many of

	will often use ChatCPT to improve my text in this way to make it easier to read."	we can use it to reflect back for our interviews. I think ultimately the ePortfolio is for us to create discussions within our groups and for ourselves and shouldn't be penalized if we use AI to organize our thoughts."	translate it). I dont think you should use it to draft the entirety of your work. If you do however, I think it penalizes the user more than anything else since they dont get that experience of critical thinking to reflect on."	my peers since I am never certain of the accuracy of information."
**34**	Respondent skipped this question	"I genuinely don't know the purpose of eportfolio. it seems to be a fruitless exercise so I see no downsides to using AI to complete, what is to many, a meaningless task."	"Maybe some guidelines on responsible use"	"I don't"
**35**	Respondent skipped this question	Respondent skipped this question	Respondent skipped this question	Respondent skipped this question
**36**	Respondent skipped this question	"The purpose is to reflect on your own experiences so not too sure why someone would need to use AI to write a self reflection"	Respondent skipped this question	Respondent skipped this question
**37**	Respondent skipped this question	Respondent skipped this question	Respondent skipped this question	"to learn new concepts, to create test material"
**38**	Respondent skipped this question	"The purpose of ePortfolio is to provide an outlet of self-réflection and understanding of the career of a doctor. I do not believ AI had a place for this as this is meant to be a reflection on personal experiences that AI can't capture."	"I think general guidelines and discussion of AI use in our introduction to ePortfolio discussion."	Respondent skipped this question
**39**	Respondent skipped this question	Respondent skipped this question	Respondent skipped this question	Respondent skipped this question
**40**	Respondent skipped this question	"eportfolio is an activity based on reflecting on experiences. AI could help in the formulation of thoughts and ideas, but the core of the reflection should be done by the writer and not replaced by AI"	"not sure"	"i use it as an adjunct study tool when reviewing theory, for example by asking for clarification or lists for certain diagnoses or testing to study for. i do not use it to produce work submitted under my name however"
**41**	Respondent skipped this question	"I do not believe that AI should be used in ePortfolio posts since it undermines the reflective learning process."	"None"	Respondent skipped this question
**42**	"I write the post myself and then have AI check the grammar and make the sentences sound more fluid"	"reflect on experiences had. AI can play a role to enhance writing and facilitate the flow of one's own ideas, however I do believe the ideas must be our own in order to be able to discuss experiences had"	Respondent skipped this question	"similar - proof read, grammar, stringing sentences together fluidly"
**43**	"I have conceptual ideas of what to	"Purpose is for reflection of our	Respondent skipped this question	"Teaching me concepts I dont

	write, but between clerkship and all of my other commitments, it can be really time consuming to type up 2 posts. ChatGPT helps start a draft and I edit it to reflect my true feelings/reflections”	experiences. AI helps document those experiences and reflections (does not replace the actual process or meaning behind ePortfolio)”		understand”
**44**	“Révision de mes textes” [Translated: “Revision of my texts.”]	Respondent skipped this question	Respondent skipped this question	Respondent skipped this question
**45**	“It helps make things easier”	“Reflection & AI can make language more eloquent”	“Nothing”	“To help with research”
**46**	Respondent skipped this question	Respondent skipped this question	Respondent skipped this question	Respondent skipped this question
**47**	Respondent skipped this question	“Encourage students to reflect on their experiences and be able to string them together into a longitudinal story that reflects their growth. I think AI can be a useful reflective tool - almost as someone to bounce ideas off of, but it should not be used to write posts in their entirety, regardless of whether the student makes edits afterwards.”	“I think the role of self-reflection should be emphasized, but strict policies won't be of much benefit - eportfolio is highly dependent on student buy-in, so strictly regulating AI usage cannot change those underlying perspectives. I also believe the monitoring and detection of AI-generated content has proved very unreliable, and may introduce unnecessary tension into the process.”	“Answering "why" questions that were not addressed in lecture (e.g: why does X condition present with X)”
